# Nonpharmacological Interventions for Pain Management in Paramedicine and the Emergency Setting: A Review of the Literature

**DOI:** 10.1155/2015/873039

**Published:** 2015-03-31

**Authors:** Sok Cheon Pak, Peter S. Micalos, Sonja J. Maria, Bill Lord

**Affiliations:** ^1^School of Biomedical Sciences, Charles Sturt University, Bathurst, NSW 2795, Australia; ^2^University of the Sunshine Coast, Sippy Downs, QLD 4556, Australia

## Abstract

Paramedicine and the emergency medical services have been moving in the direction of advancing pharmaceutical intervention for the management of pain in both acute and chronic situations. This coincides with other areas of advanced life support and patient management strategies that have been well researched and continue to benefit from the increasing evidence. Even though paramedic practice is firmly focused on pharmacological interventions to alleviate pain, there is emerging evidence proposing a range of nonpharmacological options that can have an important role in pain management. This review highlights literature that suggests that paramedicine and emergency medical services should be considering the application of complementary and alternative therapies which can enhance current practice and reduce the use of pharmacological interventions.

## 1. Introduction

Pain is a common complaint among patients cared for by paramedics [1]. Cases attended by paramedics involve patients who report pain as their chief complaint and symptom that instigated an ambulance call for assistance. In other cases, the sensation of pain will be a component of a constellation of symptoms, and the patient's report of pain will be an important diagnostic cue that guides the clinical examination. Paramedics will also encounter patients who report persistent pain, but where the pain is unrelated to their current health crisis.

Paramedics have an important role in identifying and reducing the burden of pain. The alleviation of pain is important from a humanitarian perspective, with freedom from pain considered as a basic human right [2]. Pain is also associated with significant morbidity, and as the study of pain evolves, the relationship between poorly managed acute pain and the development of chronic pain syndromes is becoming recognized [3].

Patients who seek medical care may understandably expect relief from pain, with a study of patients presenting to an emergency department finding a majority that expected relief from their pain, with a significant proportion expecting complete relief [4]. Regardless of the health care setting, pain is a frequently reported symptom. For paramedics, an encounter with a patient reporting pain is a common event [5].

The provision of reassurance and comfort for the relief of pain and distress has been described as a primary goal of paramedics and emergency medical services (EMS) [6]. However, reassurance alone may provide inadequate relief of pain. Prior to the introduction of advanced levels of training and clinical guidelines for the administration of analgesics, the management of pain in patients who were injured relied on techniques such as splinting fractures so that the immobilized limb was less likely to move and exacerbate tissue injury resulting in further pain. While there are still rudimentary skills used, paramedic practice has advanced and become more specialised and now includes the administration of a range of pharmacological agents to relieve or minimize pain [7, 8]. These now include opioids, nonsteroidal anti-inflammatories, paracetamol, NMDA-receptor antagonists, methoxyflurane, and local anaesthetics for nerve blocks. Morphine is commonly used for the treatment of pain, and this drug is considered the “gold standard” against which other analgesics are measured [9]. The efficacy of opioids such as morphine and fentanyl for the management of severe pain in the paramedic practice setting has been established [10].

Over the last two decades, this escalating reliance upon pharmaceuticals for pain management practice has been borne in part by the need to respond to societal expectations. In addition, pain management has been identified as a key performance indicator by some EMS. In Australia, the Council of Ambulance Authorities (CAA) has identified that the quality of pain relief is a surrogate measure of compassion and caring and has recently recommended that EMS develop and adopt clinical performance indicators that include the reduction of pain [22]. However, this is not a binding recommendation and national data relating to the adoption of pain management performance indicators by Australian EMS is not widely available.

The acknowledgement of pain management as an important component of paramedic practice is reflected by the use of evidence-based guidelines for the relief of pain. However, these almost exclusively focus on acute pain and pharmacological interventions. References to nonpharmacological therapies in Australian clinical guidelines for paramedics are uncommon, with the exception of traditional measures such as splinting, cooling, and reassurance. References to complementary and alternative therapies such as acupuncture are rare in the paramedic literature and resources that support paramedic education [23, 24]. Although uncommon in Australian paramedic curricula, nonpharmacological therapies for pain relief feature consistently in the practice of several allied health disciplines, with cognitive-behavioral and complementary therapies included in the International Association for the Study of Pain Core Curriculum for Health Professionals [25].

Nonpharmacological interventions to alleviate pain rely on the inhibition of pain signalling. Pain arises from nociceptive transmission through small afferents to the spinal cord and then to higher brain nuclei and the cerebral cortex. Nociceptive signals are mediated by peripheral and central components that may facilitate or inhibit this input [26]. These signals are modulated by midbrain networks which exert bidirectional control over nociceptive transmission through the spinal cord. Several neurotransmitters are involved in mediating nociceptive signals including substance P which facilitates transmission and endogenous opioid-based compounds that inhibit transmission [27]. Nonpharmacological analgesia therefore involves the inhibition of nociceptive input by activating separate antinociceptive outputs. Procedures such as transcutaneous electrical nerve stimulation (TENS) and acupoint stimulation rely on inhibiting the nociceptive signal to induce an analgesic effect.

Nonpharmacological approaches to the relief of pain are more commonly associated with nonacute settings and may be classified as follows:

(i) psychological interventions (including distraction, stress management, hypnosis, and other cognitive-behavioral interventions),
(ii) acupuncture and acupressure,
(iii) transcutaneous electrical nerve stimulation,
(iv) physical therapies (including massage, heat/cold, physiotherapy, osteopathy, and chiropractic).

These approaches to pain management may complement or indeed substitute pharmacological therapy in some types of pain. Chronic pain (which is also commonly encountered in paramedic practice) is one situation where a range of interventions may be used to manage complex health problems such as cancer pain, lower back pain, and specific diseases associated with pain such as endometriosis. Evidence of efficacy is variable, and this may be due to the type of pain, type of intervention, patient characteristics, skill and experience of the clinician, and heterogeneous study populations. For example, significant variability in the efficacy of acupuncture has been reported in the literature [28].

The use of these therapies to manage acute pain, such as pain arising from trauma or tissue injury associated with inflammation or ischemia, is rarely described in the literature. The role of alternatives in pharmacotherapy is acknowledged by the Australian and New Zealand College of Anaesthetists (ANZCA), albeit as adjunctive or complementary therapy [29]. When describing pain management in the emergency health setting, the ANZCA recommends “ice, elevation, and splinting for injuries” as well as reassurance as the mainstays of nonpharmacological management of pain [29].

This paper will appraise the current evidence of nonpharmacological interventions for pain management in the paramedic practice setting, either as complementary therapies or as alternatives to pharmacological interventions. Our review will focus upon acupuncture and acupressure, TENS, and the use of warming as all simple measures that may be implemented and would potentially complement current paramedic pain management guidelines. This contributes to the knowledge base for paramedic pain management practice and should inform future research that seeks to establish the role for nonpharmacological therapies in the relief of pain. For data sources, electronic literature searches were conducted using Medline, Embase, the Cochrane Library, and Cinahl (EBSCO). The search terms used were “paramedic” OR “CAM” OR “acupuncture” OR “acupressure” OR “TENS.”

## 2. Transcutaneous Electrical Nerve Stimulation (TENS)

Alternative approaches to paramedic-initiated analgesia such as TENS have been reported in the literature [30, 31]. However, research into the use of nonpharmacological interventions in paramedic practice is limited. This lack of research may reflect the developing status of paramedic practice as an allied health profession. There may also be limited impetus for research in this area if nonpharmacological interventions are deemed to be inappropriate for the management of pain associated with acute trauma or health emergencies, particularly in an environment where the time taken for each interval in the patient care process is a closely monitored performance indicator. The drive to minimize time spent with each patient is designed to improve operational effectiveness, and this may restrict the use of nonpharmacological therapies that require extended time to deliver the care compared with the intravenous titration of opioids. Furthermore, attitudes among paramedics and service providers regarding the utility of nonpharmacological interventions to relieve pain may inhibit clinical trials that compare the efficacy of these therapies.

Although TENS has been clinically used for over three decades, the mechanisms by which analgesia is produced are only recently being described [32]. Gate control theory is the most common theory used to support the effect of inhibiting pain by TENS. Gate control theory describes how a stimulus that activates nonnociceptive fibers can inhibit pain. Pain is reduced when the area is rubbed or stimulated due to activation of nonnociceptive fibers inhibiting the nociceptive response in the dorsal horn of the spinal cord [33]. In TENS, nonnociceptive fibers are selectively stimulated with electrodes in order to produce this effect and thereby inhibit pain [33].

TENS appears to produce both segmental and descending pain inhibition since inhibition remains after spinalization (removal of descending inhibition) in the animal model [34]. Adenosine also appears to play a role in TENS analgesia since caffeine (adenosine receptor antagonist) significantly reduces the analgesic effect resulting from activation of large diameter fibers [35]. Additionally, concentrations of endogenous opioids have been shown to increase in cerebrospinal fluid following TENS procedure [36].

TENS uses electric current produced by a portable device to stimulate the nerves for therapeutic purposes. Previous intervention trials investigating the effect of TENS on pain are shown in Table 1. One randomized double-blinded study investigating TENS in an EMS setting showed that TENS intervention in female patients (*n* = 29) with acute pelvic pain (salpingitis, ovarian cyst, dysmenorrhea, vaginal infection, and vaginal trauma) reduced pain, anxiety, heart rate, nausea, and arteriolar vasoconstriction with an improvement of overall patient satisfaction compared with those (*n* = 33) treated with sham TENS [11]. The effect of TENS producing pain relief was further supported in another study in which patients suffering from acute posttraumatic hip pain felt less pain and anxiety with TENS intervention compared with those treated with sham TENS [12]. These observations suggest that TENS could be an effective and fast-acting pain treatment with applications within paramedic practice.

## 3. Acupuncture, Electroacupuncture, and Acupressure

Stimulation of specific points on the body, commonly known as acupuncture, is widely recognized as a therapeutic procedure used to treat pain and illness [37, 38]. Acupoint stimulation such as manual acupuncture involves the penetration and manipulation of a fine needle through the skin into specified points on the body to evoke a sensation known as* de-qi* [39]. Treatment procedures that involve acupoint stimulation also include electroacupuncture and acupressure. Electroacupuncture requires delivery of electrical current through the inserted needle. Acupressure requires the use of fingers and hands to stimulate acupoints on the body to relieve pain and clinical symptoms [40]. More than 360 acupoints are located along 14 meridian channels that cover the body in a weblike interconnecting matrix [41]. Each acupoint is recognized as having a defined therapeutic action; however, a combination of acupoints is often stimulated to induce a therapeutic effect [41].

The potential mechanism for acupuncture analgesia is via the descending pain modulation pathways. The nucleus raphe magnus (NRM) in the midbrain is a significant neural site for descending analgesia via expression of serotonin [42]. NRM inhibits ascending pain signalling by projection neurons to the dorsal horn of the spinal cord. The NRM is part of a central pain modulatory system comprising the midbrain periaqueductal grey (PAG) and the ventromedial medulla (RVM) recruited to suppress or facilitate responses to noxious stimuli (PAG-RVM system). Endogenous opioid peptides are present in the neural soma and terminals of these nuclei. Electroacupuncture has been shown to enhance the expression of serotonin and reduce the release of substance P during electroacupuncture inhibition of acute nociceptive responses [43]. The dorsolateral pontine tegmentum is another midbrain site mediating spinal cord nociceptive signalling by providing noradrenergic innervations of the spinal cord. Involvement of noradrenergic receptors in rat spinal cord has also been demonstrated during electroacupuncture analgesia [44]. Together, these studies suggest that acupuncture evokes central pathways to inhibit the pain signal.

The World Health Organisation recommends the use of acupuncture for a substantial number of diseases [37]. The efficacy of acupuncture is formally endorsed by the National Institute of Health and recognized by the American Medical Association [45]. In a Cochrane Review paper on the efficacy of acupuncture for lower back pain, the results showed that acupuncture is more effective for pain relief than no treatment or sham procedure [46]. Moreover, when acupuncture is added with conventional therapy, it was shown to relieve pain and enhance function better than conventional therapies alone.

Acupressure works with the same acupoints and meridians as acupuncture. The only difference between two interventions is that acupressure stimulates the acupoints with finger pressure rather than by fine needles. Previous intervention trials investigating the effect of acupressure on pain are shown in Table 2. In the first known study to investigate the effects of acupressure on pain in the paramedic practice setting, researchers allocated adult patients (*n* = 60) to one of three treatment arms. Group 1 used true acupressure points LI4 (Hegu), PC9 (Zhongchong), PC6 (Neiguan), BL60 (Kunlun), and GV20 (Baihui), while Groups 2 and 3 involved sham acupressure point and no acupressure, respectively [13]. Group 1 patients reported reduced pain and anxiety with these changes which was significantly greater than either Group 2 or Group 3. Group 1 patients also demonstrated a statistically significant decrease in heart rate compared with patients that were treated with sham acupressure (*n* = 20) or did not receive the intervention (*n* = 21). Patient satisfaction scores after treatment were significantly better in Group 1.

In a review paper on acupressure, it was shown that this procedure was effective for pain in patients with dysmenorrhea, during labour, and in trauma [47]. In accord with this, a randomized double-blinded study in 15 patients with distal radius fracture showed that acupressure on GV20 and LI4 lowered pain, anxiety, and heart rate and raised patient general satisfaction [17]. These findings suggest that stimulation with fingers on GV20 and LI4 may be a pain management option for patients with minor trauma during ambulance transportation to a hospital. Promoting and encouraging acupressure on other acupoints that fit within the context of analgesia may create a supportive environment of pain management and is possibly an easy skill to teach all levels of paramedics.

The related technique of auricular acupressure treats the entire body through pressure on a few points in the ear. In a randomized controlled study of 36 patients with gastrointestinal illnesses (gastritis, cholecystitis, pancreatitis, and diverticulitis), researchers compared acupressure in the ear with small plastic ball at the relaxation point with a sham intervention [14]. Although both interventions showed no significant changes in blood pressure and heart rate, greater improvements in anxiety and anticipated perception of hospital treatment were reported with acupressure.

A small, randomized study showed that acupressure in the ear with 1 mm acupressure plastic beads reduced the level of pain and anxiety as indicated by a reduced heart rate in patients (*n* = 18) with hip fracture compared with patients (*n* = 20) in the sham group [16]. These observations suggest that the application of pressure to auricular acupoints may offer benefits for improving pain and anxiety. Comparable to auricular acupressure, Korean hand acupuncture with hand patches consisting of a hard plastic ball was also effective in producing analgesia [15]. A randomized study involving 100 patients with minor trauma was conducted, with the groups divided evenly (*n* = 50 per group) into intervention and sham acupressure groups. Significant improvements in nausea score, vasoconstriction, and overall patient satisfaction were achieved with Korean hand acupuncture bilaterally on K-K9 point located in the middle phalanx of the fourth finger. These simple techniques could be quite easily taught to paramedic clinicians and we would propose further experimental studies in paramedic practice. The ear is usually an area that is not injured and is out of the way of body limbs, and gaining access to apply acupressure should not be hampered by the condition of the patient. The use of acupoint pressure on the ear may prove easily accessible in a range of situations that may have positive effects which assist in relieving pain.

## 4. Effect of Warming Interventions on Pain

Another type of intervention that may be implemented by paramedics for pain control in specific situations is active warming or resistive heating, and this has been examined in several studies (Table 3). As opposed to passive heating, in which there is no external source of heat used other than the person's own body heat, active or resistive heating involves using an external source of heat to warm the patient. This may be in the form of a heated blanket or increased ambient temperature. A single-blinded randomized study reported that fifty patients undergoing active warming with minor trauma including limited bleeding, fractures, or contusions experienced less pain and anxiety with increased overall patient satisfaction, thermal comfort, and core temperature compared with another fifty undergoing passive warming [18]. A subsequent study, involving patients with a diagnosis of cholelithiasis, showed that warming with an electric heating blanket over the abdomen reduced pain, anxiety, and heart rate [19]. The subcutaneous temperature increased accordingly with increasing skin temperature. Another study using this technique published by the same group of researchers showed that patients complaining of abdominal pain from renal colic experienced less pain, anxiety, nausea, and heart rate with overall improvement of patient satisfaction [20].

Lastly, in a study of female patients with pelvic pain from cystitis, urolithiasis, cholelithiasis, appendicitis, colitis, and rectal trauma, active warming over the abdomen caused less pain, anxiety, and nausea, compared to passive warming [21]. These suggest that active warming could be an adjunct to analgesic treatment at the emergency site.

## 5. Conclusion

There are many reasons why paramedicine and emergency care practice has been moving in the direction of advanced pharmacological interventions for the management of pain in both acute and chronic situations. This coincides with other areas of advanced life support and patient management strategies that have been well researched and continue to benefit from the increasing evidence. Even though paramedic practice is firmly focused on pharmacological interventions to alleviate pain, there is a developing literature suggesting that a range of nonpharmacological options may also have an important role in managing pain in individuals cared for by paramedics.

As a developing profession, paramedicine should investigate multiple modalities and consider complementary and alternative therapies that could be used to enhance pain relief and potentially also reduce the reliance on pharmacological interventions as the first-line approach to alleviating pain. If proven to be efficacious, the analgesic sparing effect may translate into cost reductions and better patient outcomes with less adverse reactions. However, further research is required to develop the level of evidence required to support changes to practice. From the research that has been conducted, we can see great potential value of conducting trials into the use of complementary therapies within paramedic practice and would strongly encourage further research specifically that looks into the use of simple techniques such as acupuncture (including electroacupuncture and acupressure), TENS, and active warming.

## Figures and Tables

**Table 1:** Intervention trials investigating the effect of TENS on pain.

Study	Design	Participants	Intervention	Outcomes
Barker et al., 2006 [11]	Randomized double-blinded	*n* = 62 (all women) with acute pelvic pain	Transcutaneous electrical nerve stimulation (TENS)	TENS: ↓pain, anxiety, heart rate, nausea, arteriolar vasoconstriction, and ↑ overall patient satisfaction
		True TENS (*n* = 29, 24.2 ± 5.1 years old)		
		Sham TENS (*n* = 33, 26.3 ± 3.8 years old)		

Lang et al., 2007 [12]	Randomized double-blinded	*n* = 63 with acute posttraumatic hip pain	Transcutaneous electrical nerve stimulation (TENS)	TENS: ↓pain, anxiety, and heart rate
		True TENS (*n* = 30, 82.2 ± 7 years old)		
		Sham TENS (*n* = 33, 79 ± 14 years old)		

**Table 2:** Intervention trials investigating the effect of acupressure on pain.

Study	Design	Participants	Intervention	Acupoints	Outcomes
Kober et al., 2002 [13]	Randomized double-blinded	*n* = 60 with minor trauma and small injuries	Acupressure points were stimulated	LI4 (Hegu), PC9 (Zhongchong), PC6 (Neiguan), BL60 (Kunlun), and GV20 (Baihui)	Acupressure: ↓pain, anxiety, heart rate, and ↑patient satisfaction scores
		Group 1 (*n* = 19, 64 ± 21 years old, true acupressure), Group 2 (*n* = 20, 78 ± 18 years old, false acupressure), and Group 3 (*n* = 21, 63 ± 21 years old, no acupressure)			

Kober et al., 2003 [14]	Randomized double-blinded	*n* = 36 with	Acupressure in the ear with a small plastic ball (1 mm diameter)	Relaxation point	Acupressure: ↓anxiety, ↑perception of hospital medical treatment
		gastrointestinal illnesses			
		Control (*n* = 19, sham, 70.0 ± 14 years old)			
		Intervention (*n* = 17, 74.9 ± 15 years old)			

Bertalanffy et al., 2004 [15]	Randomized double-blinded	*n* = 100 with minor trauma	Korean hand acupuncture with hand patches consisting of hard plastic ball	K-K9 point located in the middle phalanx of fourth finger	Hand acupuncture: ↓nausea score, heart rate, vasoconstriction, and ↑overall patient satisfaction
		Control (*n* = 50, sham, 85.4 ± 17.1 years old)			
		Intervention (*n* = 50, 87.6 ± 16.9 years old)			

Barker et al., 2006 [16]	Randomized double-blinded	*n* = 38 with hip fracture Age = 86.2 years	Acupressure in the ear with 1 mm acupressure plastic beads	Shenmen, hip, and valium point	Acupressure: ↓pain, anxiety, and heart rate
		Control (*n* = 20, sham)			
		Intervention (*n* = 18)			

Lang et al., 2007 [17]	Randomized double-blinded	*n* = 31 with distal radius fracture Control (*n* = 16, sham) Intervention (*n* = 15)	Acupressure in the body with finger	Intervention: GV20 (Baihui) and LI4 (Hegu) Sham: BL17 (Geshu) and TE14 (Jianliao)	Acupressure: ↓pain, anxiety, heart rate, and ↑overall patient satisfaction

**Table 3:** Intervention trials investigating the effect of active warming on pain.

Study	Design	Participants	Intervention	Outcomes
Kober et al., 2001 [18]	Randomized single-blinded	*n* = 100 with minor trauma	Warming with an electric heating blanket	Resistive heating: ↓pain, anxiety, ↑overall patient satisfaction, thermal comfort, and core temperature
		Resistive heating (*n* = 50, 53–67 years old)		
		Passive warming (*n* = 50, 50–64 years old)		

Kober et al., 2003 [19]	Randomized single-blinded	*n* = 60 with cholelithiasis	Warming with an electric heating blanket over abdomen	Active warming: ↓pain, anxiety, heart rate, and ↑skin and subcutaneous temperature
		Active warming (*n* = 30, 47.8 ± 18.2 years old)		
		Passive warming (*n* = 30, 42.9 ± 21.0 years old)		

Kober et al., 2003 [20]	Randomized single-blinded	*n* = 100 with acute renal colic	Warming with an electric blanket set to 42°C	Resistive heating: ↓pain, anxiety, nausea, vasoconstriction, heart rate, and ↑overall patient satisfaction
		Resistive heating (*n* = 36, 27.6 ± 6.8 years old)		
		Passive warming		
		(*n* = 38, 29.4 ± 7.1 years old)		

Bertalanffy et al., 2006 [21]	Randomized single-blinded	*n* = 62 (all women) with pelvic pain	Warming with an electric heating blanket over abdomen	Active warming: ↓pain, anxiety, nausea, heart rate, and vasoconstriction
		Active warming (*n* = 29, 24.2 ± 5.1 years old)		
		Passive warming (*n* = 33, 26.3 ± 3.8 years old)		

## References

[1] Bendall J. C., Simpson P. M., Middleton P. M. (2011). Prehospital analgesia in New South Wales, Australia. *Prehospital and Disaster Medicine*.

[2] Cousins M. J., Lynch M. E. (2011). The Declaration Montreal: access to pain management is a fundamental human right. *Pain*.

[3] Voscopoulos C., Lema M. (2010). When does acute pain become chronic?. *British Journal of Anaesthesia*.

[4] Fosnocht D. E., Heaps N. D., Swanson E. R. (2004). Patient expectations for pain relief in the ED. *The American Journal of Emergency Medicine*.

[5] Jennings P. A., Cameron P., Bernard S. (2011). Epidemiology of prehospital pain: an opportunity for improvement. *Emergency Medicine Journal*.

[6] Callaham M. (1997). Quantifying the scanty science of prehospital emergency care. *Annals of Emergency Medicine*.

[7] Ambulance Victoria (2012). *Clinical Practice Guidelines for MICA and Ambulance Paramedics*.

[8] Queensland Ambulance Service (2012). *QAS Clinical Practice Manual*.

[9] Lugo R. A., Kern S. E. (2002). Clinical pharmacokinetics of morphine. *Journal of Pain & Palliative Care Pharmacotherapy*.

[10] Middleton P. M., Simpson P. M., Sinclair G., Dobbins T. A., Math B., Bendall J. C. (2010). Effectiveness of morphine, fentanyl, and methoxyflurane in the prehospital setting. *Prehospital Emergency Care*.

[11] Barker R., Lang T., Steinlechner B. (2006). Transcutaneous electrical nerve stimulation as prehospital emergency interventional care: treating acute pelvic pain in young women. *Neuromodulation*.

[12] Lang T., Barker R., Steinlechner B. (2007). TENS relieves acute posttraumatic hip pain during emergency transport. *The Journal of Trauma—Injury, Infection and Critical Care*.

[13] Kober A., Scheck T., Greher M. (2002). Prehospital analgesia with acupressure in victims of minor trauma: a prospective, randomized, double-blinded trial. *Anesthesia and Analgesia*.

[14] Kober A., Scheck T., Schubert B. (2003). Auricular acupressure as a treatment for anxiety in prehospital transport settings. *Anesthesiology*.

[15] Bertalanffy P., Hoerauf K., Fleischhackl R. (2004). Korean hand acupressure for motion sickness in prehospital trauma care: a prospective, randomized, double-blinded trial in a geriatric population. *Anesthesia and Analgesia*.

[16] Barker R., Kober A., Hoerauf K. (2006). Out-of-hospital auricular acupressure in elder patients with hip fracture: a randomized double-blinded trial. *Academic Emergency Medicine*.

[17] Lang T., Hager H., Funovits V. (2007). Prehospital analgesia with acupressure at the Baihui and Hegu points in patients with radial fractures: a prospective, randomized, double-blind trial. *The American Journal of Emergency Medicine*.

[18] Kober A., Scheck T., Fülesdi B., Liera F., Vlach W., Friedman A. (2001). Effectiveness of resistive heating compared with passive warming in treating hypothermia associated with minor trauma: a randomized trial. *Mayo Clinic Proceedings*.

[19] Kober A., Scheck T., Tschabitscher F. (2003). The influence of local active warming on pain relief of patients with cholelithiasis during rescue transport. *Anesthesia and Analgesia*.

[20] Kober A., Dobrovits M., Djavan B. (2003). Local active warming: an effective treatment for pain, anxiety and nausea caused by renal colic. *Journal of Urology*.

[21] Bertalanffy P., Kober A., Andel H., Hahn R., Frickey N., Hoerauf K. (2006). Active warming as emergency interventional care for the treatment of pelvic pain. *BJOG: An International Journal of Obstetrics & Gynaecology*.

[22] Council of Ambulance Authorities (2001). *Clinical Performance Indicators. Indicator Area 5: Trauma Pain Relief*.

[23] Lord B. (2009). Book review: paramedic practice today: above and beyond. *Journal of Emergency Primary Health Care*.

[24] Curtis K., Ramsden C., Lord B. (2011). *Emergency and Trauma Care: For Nurses and Paramedics*.

[25] Charlton J. E. (2005). *Core Curriculum for Professional Education in Pain*.

[26] Fields H. L., Basbaum A. I., Wall P. D., Melzack R. (1999). Central nervous system mechanisms of pain modulation. *Textbook of Pain*.

[27] Ren K., Dubner R., Basbaum A. I., Bushnell M. C. (2009). Descending control mechanisms. *Science of Pain*.

[28] Lee M. S., Ernst E. (2011). Acupuncture for pain: an overview of Cochrane reviews. *Chinese Journal of Integrative Medicine*.

[29] Macintyre P. E., Schug S. A., Scott D. A., Visser E. J., Walker S. M. (2010). *Acute Pain Management: Scientific Evidence*.

[30] Mora B., Giorni E., Dobrovits M. (2006). Transcutaneous electrical nerve stimulation: an effective treatment for pain caused by renal colic in emergency care. *The Journal of Urology*.

[31] Simpson P. M., Fouche P. F., Thomas R. E., Bendall J. C. (2014). Transcutaneous electrical nerve stimulation for relieving acute pain in the prehospital setting: a systematic review and meta-analysis of randomized-controlled trials. *European Journal of Emergency Medicine*.

[32] Sluka K. A., Walsh D. (2003). Transcutaneous electrical nerve stimulation: basic science mechanisms and clinical effectiveness. *The Journal of Pain*.

[33] Kandel E. R., Schwartz J. H., Jessell T. M. (2000). *Principles of Neural Science*.

[34] Woolf C. J., Mitchell D., Barrett G. D. (1980). Antinociceptive effect of peripheral segmental electrical stimulation in the rat. *Pain*.

[35] Marchand S., Li J., Charest J. (1995). Effects of caffeine on analgesia from transcutaneous electrical nerve stimulation. *The New England Journal of Medicine*.

[36] Salar G., Job I., Mingrino S., Bosio A., Trabucchi M. (1981). Effect of transcutaneous electrotherapy on CSF *β*-endorphin content in patients without pain problems. *Pain*.

[37] World Health Organization (2003). *Acupuncture Review and Analysis of Reports on Controlled Clinical Trials*.

[38] He L. F. (1987). Involvement of endogenous opioid peptides in acupuncture analgesia. *Pain*.

[39] Lin J. G., Chen W. L. (2009). Review: acupuncture analgesia in clinical trials. *The American Journal of Chinese Medicine*.

[40] Chen Y.-W., Wang H.-H. (2014). The effectiveness of acupressure on relieving pain: a systematic review. *Pain Management Nursing*.

[41] Kaptchuk T. J. (2002). Acupuncture: theory, efficacy, and practice. *Annals of Internal Medicine*.

[42] Fields H. L., Basbaum A. I., Heinricher M. M., McMahon S. B., Koltzenburg M. (2006). Central nervous system mechanisms of pain modulation. *Wall and Melzack’s Textbook of Pain*.

[43] Yonehara N. (2001). Influence of serotonin receptor antagonists on substance P and serotonin release evoked by tooth pulp stimulation with electro-acupuncture in the trigeminal nucleus cudalis of the rabbit. *Neuroscience Research*.

[44] Sun K. K., Jung H. P., Sang J. B. (2005). Effects of electroacupuncture on cold allodynia in a rat model of neuropathic pain: mediation by spinal adrenergic and serotonergic receptors. *Experimental Neurology*.

[45] Ramsay D. J., Bowman M. A., Greenman P. E. (1998). Acupuncture. *Journal of the American Medical Association*.

[46] Furlan A. D., van Tulder M. W., Cherkin D. C. (2005). Acupuncture and dry-needling for low back pain. *The Cochrane Database of Systematic Reviews*.

[47] Lee E. J., Frazier S. K. (2011). The efficacy of acupressure for symptom management: a systematic review. *Journal of Pain and Symptom Management*.

